# Ocular Disorders in Adult Patients with Tuberculosis in a Tertiary Care Hospital in Nigeria

**DOI:** 10.4103/0974-9233.51996

**Published:** 2008

**Authors:** E.E. Egbagbe, A.E. Omoti

**Affiliations:** From the Department of Medicine, University of Benin Teaching Hospital, Benin City, Nigeria

**Keywords:** ocular, visual loss, tuberculosis, adult, uveitis, Nigeria

## Abstract

**Objectives:**

To determine the ocular disorders in adult tuberculosis (TB) patients in Benin City, Nigeria.

**Methods:**

A prospective study of adult TB patients presenting at the University of Benin Teaching Hospital, Benin City, Nigeria, between March 2006 and October 2006 was undertaken. The patients were interviewed and examined by the authors and the ocular findings recorded.

**Results:**

There were 92 patients (45 males and 47 females) with mean age 37.9 years (SD±15.6). Only one (1.1 percent) was HIV positive. Among the ocular findings in patients with TB, 8 patients had monocular blindness that included cataracts in 3 (37.5 percent), glaucoma in 2 (25 percent), optic atrophy, retinal vasculitis and maculopathy accounting for one case each (12.5 percent). Ocular disorders due to TB occurred in 9 patients (9.8 percent). These include cataract in 2 cases (2.2 percent), phlyctenular conjunctivitis in 2 cases (2.2 percent), glaucoma, anterior uveitis, chorioretinitis, retinal vasculitis, maculopathy, and optic atrophy each occurring in 1 case (1.1 percent).

**Conclusion:**

Tuberculosis is a cause of ocular morbidity, visual impairment and blindness. Prevention, early diagnosis and early treatment of TB may prevent avoidable visual loss.

Tuberculosis (TB) is regarded as one of the oldest diseases in the world. It is a chronic infectious disease caused by Mycobacterium tuberculosis, which is a member of a group of closely related organisms in the M. tuberculosis complex (Mycobaclorium africanum, Mycobacterium bovis, Mycobacterium mictroti and Mycobacterium tuberculosis).^1^ Worldwide, there are approximately 8-10 million new cases of TB every year, 95 percent in developing countries.^2^ Nearly 3 million people die from TB each year; 98 percent of infection-related deaths occurring in the developing world.^2^

Ocular TB involving any of the tissues of the eye is uncommon.^3^ Dutt et al^4^ reported ophthalmic involvement in only 3 of 402 sites of extrapulmonary TB. Donahue^5^ reported that 1.4 percent of more than 10,000 patients in one sanatorium were treated for ocular TB between 1940 and 1966. In Nigeria, Ayanru^6^ reported that only one case out of 1987 cases of uveitis was due to tuberculosis. Tuberculosis accounted for less than 1 percent of uveitis in another study in Los Angeles.^7^ Donahue^5^ found only 6 cases of phlyctenular conjunctivitis in 10,524 patients with TB.

The impact of the AIDS epidemic on rates of ocular tuberculosis remains unclear. With the recent increase in incidence of TB in both the general population and AIDS patients, ocular manifestations of TB once thought to be rare may be increasing.^8^

This study was designed to identify ocular disorders in adult TB patients attending the consultant outpatient clinic at the University of Benin Teaching Hospital, Benin City, Nigeria.

## Materials and Methods

Adult tuberculosis patients attending the consultant outpatient medical clinic of the University of Benin Teaching Hospital – a tertiary care center in Benin City, Nigeria, between March and October 2006 were interviewed by the authors using a standard questionnaire and were examined in the eye clinic.

The visual acuity was determined using the standard illuminated Snellen's chart or illiterate E chart held at 6 meters from the patient. When the patient could not read the chart, the ability to count fingers at varying distances, to perceive hand movements or light was determined and recorded. The eyes were examined using a pentorch, Haag Streit slitlamp biomicroscope and the direct ophthalmoscope. The intraocular pressures were measured by using the Goldmann applanation tonometer mounted on the Haag Streit slitlamp. Indirect ophthalmoscopy was performed when there was evidence or suspicion of retinal lesions.

All patients were diagnosed as having TB based on results of chest radiograph, sputum examination (presence of acid fast bacilli on 3 samples) and in cases of extrapulmonary tuberculosis, histological findings suggestive of tuberculosis. All the patients were screened for HIV using ELISA technique and positive cases were confirmed using the Western blot technique.

## Results

Ninety-two patients (45 males and 47 females) were seen during the period of study. The age and sex distribution of the patients is shown in Table 1. The mean age was 37.9 years (SD±15.6).

**Table 1:** Age and Sex Distribution of Patients with Tuberculosis (n = 92)

Age	Sex		(%)
		
	Male (45)	Female (47)	
<20 yrs	2	5	7 (7.6)

21-30	15	16	31 (33.7)

31-40	8	7	15(16.3)

41-50	7	5	12(13.1)

51-60	9	6	15(16.3)

61-70	1	6	7(7.6)

71 and above	3	2	5 (5.4)

Fourteen patients (15.2 percent) were illiterates, 15 patients (16.3 percent) had primary education, 36 patients (39.1 percent) had secondary education and 27 patients (29.3 percent) had tertiary education. The occupation of the patients was classified according to a modified form of the British Registrar Generals classification.^9^ There were 3 patients (3.3 percent) in the higher professions, who were all engineers. Six patients (6.5 percent) were in the other professions such as teachers and shopkeepers. There were 25 skilled workers (27.2 percent) such as clerks, electricians and drivers. There were 14 semiskilled workers (15.2 percent) such as machine operators and 12 unskilled workers (13 percent) such as farmers and laborers. Among others, there were 29 students (31.5 percent) and 3 unemployed (3.3 percent).

Forty-eight patients (52.1 percent) had been newly diagnosed with TB, 41 patients (44.6 percent) had TB for 5 years or less, 2 (2.2 percent) for 6 to 10 years and one patient (1.1 percent) for more than 10 years.

The presenting ocular complaints of patients with tuberculosis are shown in Table 2. Of those with ocular symptoms, 4 patients (16.7 percent) had ocular symptoms for less than one month, 5 (20.8 percent) for one to six months, 5 (20.8 percent) for 7 to 12 months, 7 (29.2 percent) for between 1 year and 5 years, and 3 patients (12.5 percent) had ocular symptoms for more than 5 years. Only one patient was positive for HIV-1. The 48 newly diagnosed patients (52.2 percent) had not been treated with antituberculosis drugs at the time of their ocular examination while the remaining 44 patients (47.8 percent) had been on antituberculosis regimen comprising rifampicin, ethambutol, isoniazid and pyrazinamide.

**Table 2:** Ocular Complaints in Patients with Tuberculosis (n = 92)

Eye Complaints	Number of Patients (%)
Poor vision	14(15.2)

Difficulty reading	5 (5.4)

Itching	2 (2.2)

Tearing	1 (1.1)

Pain	1 (1.1)

Peppery sensation	1 (1.1)

Sixty-five patients (70.7 percent) had visual acuity of 6/5 – 6/6, 19 patients (20.6 percent) had visual acuity of 6/9 – 6/12 while 8 patients (8.7 percent) had best corrected vision of 6/18 – 6/24. Monocular blindness (inability to count fingers at 3 meters in the eye) occurred in 8 patients (8.7 percent). It was due to cataract in 3 cases (37.5 percent), glaucoma in 2 cases (25 percent), optic atrophy in one case (12.5 percent), retinal vasculitis in one case (12.5 percent) and maculopathy in one case (12.5 percent).

The intraocular pressures ranged from 8mmHg to 36mmHg. The mean intraocular pressure was 15.3mmHg (SD±4.6mmHg). Only 7 eyes in 4 patients had initial intraocular pressures greater than 21mmHg. All the ocular disorders seen in patients are shown in Table 3. The ocular disorders due to tuberculosis are shown in Table 4. These disorders occurred in 9 patients (9.8 percent). The case of maculopathy occurred in the same patient with the retinal vasculitis.

**Table 3:** Ocular Findings in Tuberculosis Patients

Ocular Findings	Number of Patients (%)
Cataract	11 (12)

Refractive error	9 (9.8)

Pterygium	9 (9.8)

Glaucoma	6 (6.5)

Presbyopia	5 (5.4)

Maculopathy	5 (5.4)

Allergic conjunctivitis	5(5.4)

Age related macula degeneration	4 (4.3)

Pingueculum	4 (4.3)

Phlyctenular conjunctivitis	2 (2.2)

Chronic anterior uveitis	1 (1.1)

Chorioretinitis	1 (1.1)

Optic atrophy	1 (1.1)

Retinal vasculitis	1 (1.1)

**Table 4:** Ocular Disorders Attributable to Tuberculosis

Ocular Disorder	Number of Patients (%)
Cataract	2 (2.2)

Glaucoma	1 (1.1)

Maculopathy	1 (1.1)

Phlyctenular conjunctivitis	2 (2.2)

Chronic anterior uveitis	1(1-1)

Chorioretinitis	1 (1.1)

Optic atrophy	1 (1.1)

Retinal vasculitis	1 (1.1)

## Discussion

Tuberculosis can affect practically any structure of the eye and adnexae.^1^ Ocular manifestations in TB may be attributed to either infection or non-infectious immunologic reactions. Haematogenous dissemination may result in involvement of the uvea because of its greater vascularity while immunological reactions to tuberculoprotein may cause phlyctenulosis, interstitial keratitis and retinal vasculitis.^1^

There was no sex predilection in the patients seen in this study and the majority of them were in the lower socioeconomic classes or students. The high proportion of students may be related to the close proximity of several tertiary institutions to the University of Benin Teaching Hospital where the study was conducted.

Over half of the patients were newly diagnosed with TB and were not on any therapy at the time of eye examination. The majority of the patients did not complain of any ocular symptoms. This is in agreement with other studies which show that ocular involvement in TB is uncommon.^3^–^7^ The majority of the ocular complaints in this study were unrelated to TB. An unexpected finding was the very low incidence of HIV seropositivity in this study. This may be because patients with an initial diagnosis of having HIV/AIDS, attend a special clinic in this hospital and not the general medical clinics in which this study was carried out.

The majority of patients had good vision in the better eye. None of the patients was blind but monocular blindness occurred in 8 patients (8.7 percent). They were due mainly to cataract and glaucoma and to a lesser extent, optic atrophy, retinal vasculitis and maculopathy. Several of these may be related to TB. Some cases of cataract may be age related, secondary to TB, anterior uveitis^1^ or due to other causes. Glaucoma may also be secondary to chronic anterior uveitis due to TB.^1^ Optic atrophy may occur due to tuberculosis, antituberculosis drugs^10^ or other causes. In TB, the optic nerve may be affected directly, as part of tuberculous posterior uveitis or through direct infiltration as part of a tuberculous meningitis.^1^ Retinal vasculitis in this study occurred in the left eye of a 29 year old student with testicular TB. It was associated with choroidal folds and macular edema. Possible causes of retinal vasculitis in patients with TB include haematogenous dissemination of infection, inflammation from adjacent chorioretinal lesions or hypersensitivity reactions.^1^,^11^ The type of maculopathy seen in this case was cystoid macula edema. Cystoid macular edema has been reported as one of the presentations of tubercular uveitis.^12^ Cystoid macular edema has also been reported as the only ocular finding in TB.^13^

The most common ocular disorders seen in the TB patients were cataract, refractive error, pterygium, glaucoma, presbyopia, maculopathy and allergic conjunctivitis. This is different from findings in hospital based studies in the same institution,^14^ and other parts of Nigeria,^15^ where refractive errors are reported to be by far the most common ocular disorders. Allergic conjunctivitis is also reported to be much more common than glaucoma and maculopathy in contrast to the finding in this study.^14^,^15^ Population based studies in the immediate vicinity of the hospital^16^ and elsewhere in Nigeria^17^ also show that refractive errors are the most common ocular disorders. These differences may be due to the effect of TB in the eyes. Tuberculosis causes chronic uveitis which may be complicated by cataract, glaucoma and cystoid macula oedema.^1^,^12^,^13^ This may have contributed to the relatively higher frequencies of these conditions in tuberculosis patients in this study. The other conditions which may have been due primarily to TB include phlyctenular conjunctivitis, chronic anterior uveitis, multifocal chorioretinitis, optic atrophy and retinal vasculitis. These conditions could be caused either by direct infiltration or hypersensitivity reaction. The one case of optic atrophy was most likely due to TB rather than the side effects of antituberculous drugs because visual loss started before commencement of therapy. One case of cystoid macula edema was associated with retinal vasculitis. Two cases of cataract and one case of glaucoma had a past history of recurrent redness of their eyes and signs suggestive of previous uveitis such as irregular pupil and iris pigment on the anterior lens capsule. Only 2 cases showed obvious signs of active uveitis, one with chronic anterior uveitis and the other with a multifocal chorioretinitis. This is similar to the findings in other reports.^6^,^7^ Multifocal choroiditis has been reported as a manifestation of ocular TB.^18^ In a similar study in Brazil, most of the ocular findings in patients with TB were in the anterior segment.^3^ This is similar to the finding in this report. Ocular TB was also shown to have a low prevalence but important visual loosing morbidity.^3^

In conclusion, TB is a cause of ocular morbidity, and visual loss. Prevention of TB, and early diagnosis and treatment may reduce ocular morbidity and prevent avoidable visual loss many of which occur from complications of chronic uveitis.
